# Pediatric-onset Multiple Sclerosis treatment: a multicentre observational study comparing natalizumab with fingolimod

**DOI:** 10.1007/s00415-024-12610-y

**Published:** 2024-08-23

**Authors:** Antonio Carotenuto, Cristina Di Monaco, Laura Papetti, Giovanna Borriello, Elisabetta Signoriello, Camilla Masciulli, Valentina Tomassini, Giovanna De Luca, Antonio Ianniello, Giacomo Lus, Federica Novarella, Antonio Luca Spiezia, Dario Di Somma, Marcello Moccia, Maria Petracca, Carmine Iacovazzo, Giuseppe Servillo, Emilio Portaccio, Maria Triassi, Maria Pia Amato, Carlo Pozzilli, Massimiliano Valeriani, Vincenzo Brescia Morra, Roberta Lanzillo

**Affiliations:** 1 Department of Neuroscience, Reproductive Science and Odontostomatology, Multiple Sclerosis Clinical Care and Research Centre, Federico II University, Naples, Italy; 2 Developmental Neurology Unit, Bambino Gesù Children’s Hospital, IRCCS, Rome, Italy; 3 Department of Public Health, University “Federico II” of Naples, Naples, Italy; 4 Second Division of Neurology, Department of Advanced Medical and Surgical Sciences, University of Campania, Naples, Italy; 5 Department of NEUROFARBA, University of Florence, Florence, Italy; 6 Multiple Sclerosis Centre, Clinical Neurology, SS. Annunziata University Hospital, Chieti, Italy; 7 Institute for Advanced Biomedical Technologies (ITAB), Department of Neurosciences, Imaging and Clinical Sciences, University G. d’Annunzio of Chieti-Pescara, Chieti, Italy; 8 Department of Human Neurosciences, Sapienza University, Rome, Italy; 9 Department of Molecular Medicine and Medical Biotechnology, Federico II University of Naples, Naples, Italy; 10 System Medicine Department, Tor Vergata University of Rome, Rome, Italy; 11 Center for Sensory-Motor Interaction, Aalborg University, Aalborg, Denmark

**Keywords:** Multiple Sclerosis, natalizumab, fingolimod, pediatric multiple sclerosis, disease modifying treatment, real-world study

## Abstract

**Background:**

Pediatric-onset Multiple Sclerosis (POMS) patients show more inflammatory disease compared with adult-onset MS. However, highly effective treatments are limited with only fingolimod being approved in Italy and natalizumab prescribed as off-label treatment.

**Objectives:**

to compare the efficacy of natalizumab versus fingolimod in POMS.

**Methods:**

This is an observational longitudinal multicentre study including natalizumab- and fingolimod-treated POMS patients (N-POMS and F-POMS, respectively). We collected Annual Relapse Rate (ARR), Expanded Disability Status Scale (EDSS), Symbol Digit Modality Test (SDMT), and MRI activity at baseline (T0), 12–18 months (T1), and last available observation (T2).

**Results:**

We enrolled 57 N-POMS and 27 F-POMS patients from six Italian MS Centres. At T0, N-POMS patients showed higher ARR (*p* = 0.03), higher EDSS (*p* = 0.003) and lower SDMT (*p* = 0.04) at baseline compared with F-POMS. Between T_0_ and T_1_ ARR improved for both N-POMS and F-POMS (*p* < 0.001), while EDSS (*p* < 0.001) and SDMT (*p* = 0.03) improved only for N-POMS. At T_2_ (66.1 ± 55.4 months) we collected data from 42 out of 57 N-POMS patients showing no further ARR decrease.

**Conclusion:**

Both natalizumab and fingolimod showed high and sustained efficacy in controlling relapses and natalizumab also associated to a disability decrease in POMS. This latter effect might be partly mediated by the high inflammatory activity at baseline in N-POMS.

## Introduction

Multiple Sclerosis (MS) is a chronic immune-mediated inflammatory disease of the central nervous system. While MS primarily affects young adults, with an incidence peak between 20 and 40 years, it presents before the age of 18 in approximately 3–10% of cases [1]. The natural history of Pediatric-Onset Multiple Sclerosis (POMS) markedly differs from that of adult-onset MS (AOMS), with POMS showing greater inflammatory activity, reflected in higher relapse rate and accelerated accumulation of new Magnetic Resonance Imaging (MRI) lesions, compared with AOMS [2–7]. Differently from AOMS, POMS cases typically exhibit a slower progression toward significant disability though they reach key disability milestones approximately 10 years earlier than AOMS patients [2].

The risk of disability decreases significantly in individuals promptly treated with high-efficacy Disease-Modifying Therapies (DMTs) [8]. The therapeutic management of patients with POMS is challenging due to the difficulty to perform randomized controlled trials assessing the efficacy and safety in children of DMTs already used to treat adult patients [9]. In Europe, fingolimod is the only approved DMT by EMA and the National Regulatory Agency (AIFA) for POMS, based on findings from PARADIGMS trial [10]. In this trial, fingolimod was proven to be effective in reducing relapse rate, new MRI lesions, and brain atrophy accrual over a 2-year period vs interferon beta 1a. Other high-efficacy DMTs (e.g., natalizumab) are prescribed exclusively as off-label treatment given the efficacy and safety profile of the drug reported from observational and prospective real-world data.

Published observational studies reporting off-label natalizumab usage consistently demonstrate the efficacy of natalizumab in reducing disease activity in POMS, including those with aggressive disease, without significant adverse effects [11–13]. However, natalizumab can be prescribed to POMS patients in whom the use of fingolimod is contraindicated, has not been tolerated or has not been shown to be effective, and with specific criteria (two or more disabling relapses in the last year, and 1 or more gadolinium-enhancing lesions on brain MRI or with a significant increase in lesion load at T2 compared to a previous MRI performed at least three months apart [14]). Furthermore, natalizumab is associated with a higher risk of progressive multifocal leukoencephalopathy, caused by the JC virus [15]. Therefore, clinicians are usually cautious to prescrive natalizumab in those patients with higher Anti-JC virus antibodies titres.

Studies comparing prescription patterns of natalizumab and fingolimod in clinical settings (i.e., patients population addressed to either fingolimod or natalizumab) or comparing treatment effectiveness are still lacking. Different real-world studies comparing long-term and short-term safety and efficacy between the two drugs in the adult population have reported contrasting results, with some studies showing a trend favoring natalizumab [16–20]. A recent study analyzing data from three MS registries showed that natalizumab had higher efficacy compared with fingolimod in AOMS patients [21]. Considering the scarcity of available data in the pediatric population, we conducted a multicentre longitudinal observational study in Italy to describe the use of available high-efficacy therapies in POMS. The objective is to evaluate clinical utilization of fingolimod and natalizumab in real-world settings and to compare the efficacy of the two drugs accounting for the possible selection biases.

## Methods

**Fig. 1:**
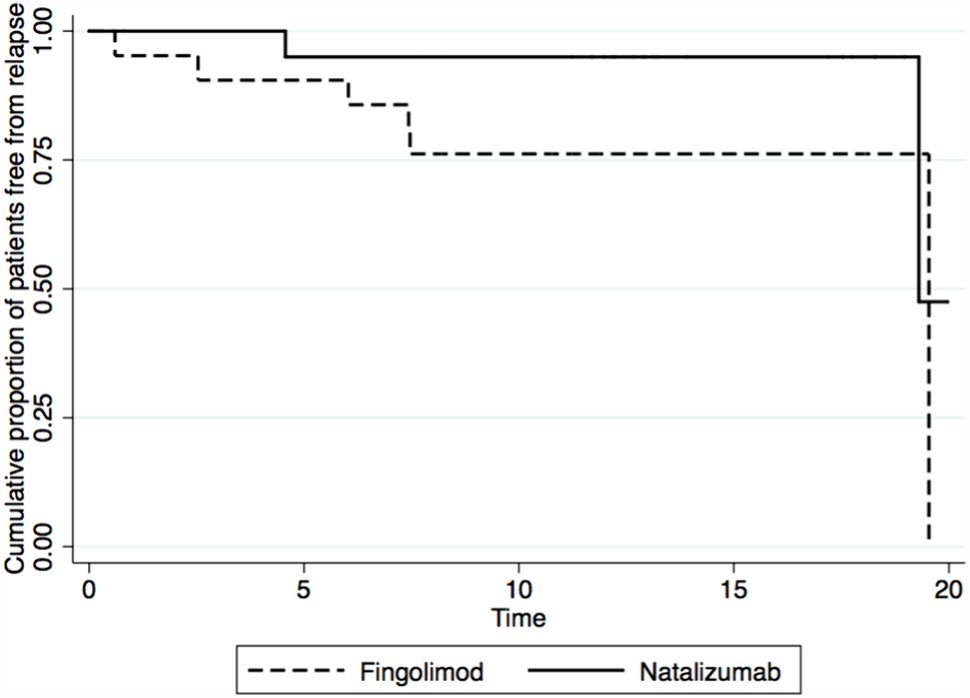
Analysis of time to relapse: Cox regression analysis after propensity score matching showing no differences in time to first relapse between N-POMS and F-POMS

This is a multicentre longitudinal retrospective study. We included POMS patients from 6 Italian MS Centres (1.Federico II University, Naples; 2. Bambino Gesù Hospital IRCCS, Rome; 3. Sapienza University of Rome, Rome; 4. University of Campania, Naples; 5.University of Florence, Florence; 6. SS. Annunziata University Hospital, Chieti), satisfying the following inclusion criteria: (1) MS diagnosis according to Krupp criteria [22] (2) POMS with treatment with natalizumab or fingolimod start before 18 years of age; (3) age at last available follow-up < 25 years old; (4) patients starting on fingolimod (F-POMS) or natalizumab (N-POMS) treatment with at least 12 month of follow-up; (5) body weight between 50 and 100 kg allowing a standard treatment dosage; (6) no other major systemic, psychiatric or neurologic diseases. POMS patients treated with both fingolimod and natalizumab in sequence were included only for the DMT started earlier (see Fig. 1).

### Standard protocol approvals, registrations, and patient consents

Approval was received from the ‘Comitato Etico Campania 3’ (approval number:0023943). All subjects and parents, when necessary, gave written informed consent prior to study participation. The study was performed in accordance with good clinical practices and the Declaration of Helsinki.

### Clinical assessment

We retrospectively included patients treated with natalizumab and fingolimod, with at least two clinical assessment 12–18 months apart: at baseline corresponding treatment start (T_0_) and after 12–18 months (T_1_). Since natalizumab has been used for longer time in clinical settings, we also collected clinical and radiologic data up to the last available assessment (T_2_) for N-POMS.

Patients treated with natalizumab received intravenous 300 mg administration of natalizumab each 28 days. Patients treated with fingolimod received oral 0.5 mg per day.

At T_0_ we recorded clinical and demographic data (mandatory data: age, sex, disease duration expressed in months, number of previous disease modifying treatments, previous DMT, reasons for switching from previous disease modifying treatments, annualized relapse rate, Expanded Disability Status Scale (EDSS) [23]; Paediatric Multiple Sclerosis Severity Score (Ped-MSSS) [24]; supplemental data: lesion load based on conventional T2-weighted MRI scan [low if < 10lesions; medium if lesions number between 10 and 25; high if lesions > 25] assessed from neurologists involved in the study with demonstrated expertise in reviewing MRI images as for conventional radiologic reporting systems [25], contrast-enhancing lesions [yes/no], symbol digit modality test (SDMT) [26], JCV positivity [yes/no]). At T_1_ and T_2_ we recorded the following clinical data: time from previous assessment, EDSS, relapse occurrence and time to relapse, occurrence of DMT switch, time to DMT switch and reason for DMT switch (inefficacy, tolerability [lack of adherence to drug administration protocol for personal choice or willingness of different mode of administration], safety concerns), lesion load based on conventional T2-weighted MRI scan (low if < 10lesions; medium if lesions number between 10 and 25; high if lesions > 25), contrast-enhancing lesions (yes/no), SDMT, JCV positivity (yes/no). MRI activity was defined based on the radiologic report provided from the radiologist blinded to patients’ treatment. MRI activity was defined either as contrast-enhancing lesion and/or new/enlarging T2 lesions at follow-up scans.

### Statistical analysis

Statistical analyses were performed using the Stata software (version 13; StataCorp LP, College Station, TX). Demographic, clinical and radiologic features of study subjects are presented as means, medians or proportions as appropriate. All demographic, clinical and laboratory variables were checked for normality using the Shapiro–Wilk normality test. Differences between F-POMS and N-POMS for demographic and clinical features at T_0_ were assessed through *t*-Test, Mann–Whitney *U* or Chi-squared as appropriate.

Paired-Samples *t* test or Wilcoxon matched-paired signed-rank test was used to compare Annual Relapse Rate (ARR), EDSS and SDMT among different time points for each drug.

We performed a propensity score (PS) matching analysis accounting for possible differences in clinic-demographic variables at T_0_ in the two arms too explore natalizumab or fingolimod associated with relapse occurrence or DMT switching over the follow-up.

Specifically, to balance observed covariates (i.e., age, sex, EDSS, Ped-MSSS, ARR and number of previous treatment) between F-POMS and N-POMS we employed the PS method. A Binary Logistic Regression Model, with treatment group as dependent variable, was applied for the calculation of the PS. The PS was calculated as the predicted probability of occurrence of the event (treatment group allocation). To obtain two groups not differing for considered covariates we performed a 1-to-1 matching with no replacement methods using a caliper of 0.2 as maximum PS score distance to match groups. We applied the matching technique instead of the adjusting PS technique as to be more conservative. Using the created groups we performed analysis of survival by Cox Regression model, for time to DMT switch and time to first relapse occurrence. A *p* value < 0.05 was considered statistically significant. Results are presented with 95% confidence interval (95%CI) or *p* values.

### Data availability

The anonymised dataset used and analyzed during the current study is available from the corresponding author upon reasonable request.

## Results

### Clinical and MRI measures at baseline

Demographic and clinical data from subjects enrolled in the study are summarized in Table 1. We included 84 MS patients (27 patients treated with fingolimod [disease duration (mean ± SD): 16.3 ± 26.1 months] and 57 patients treated with natalizumab [disease duration: 13.5 ± 15.4 months]). Compared with F-POMS, N-POMS showed higher number of total relapse before T_0_ (median [range]: 2 [0–10] vs 1 [1–6], *p* = 0.02), higher ARR before T_0_ (1 [0–5.2] vs 1 [0.1–4.4], p = 0.03), higher EDSS at T_0_ (median [range]: 2 [0–6] vs 1.5 [0–3.5], *p* = 0.003), higher Ped-MSSS [mean ± SD: 7.2 ± 2.9 vs 5.2 ± 3.2, *p* = 0.005], higher prevalence of patients switching from previous DMT due to inefficacy (72% vs 37%, *p* = 0.02) and lower SDMT ((mean ± SD): 49.4 ± 7.9 vs 60.2 ± 9.2, *p* = 0.04).

**Table 1:** Demographic and clinical features for the enrolled patients

	Fingolimod	Natalizumab	*p* value
Subjects	27	57	
Center			
Federico II University, *N* (%)	7 (26)	20 (35)	
University of Florence, *N* (%)	2 (7)	2 (3)	
University of Campania, *N* (%)	4 (15)	6 (10)	
Hospital Bambino Gesù, *N* (%)	9 (34)	18 (32)	
University of Rome ‘La Sapienza’, *N* (%)	2 (7)	9 (17)	
Sant' Annunziata University Hospital, *N* (%)	3 (11)	2 (3)	
Sex			
Female, *N* (%)	19 (70)	40 (70)	0.98
Male, *N* (%)	8 (30)	17 (30)	
Age, mean ± SD (years)	16.2 ± 2.7	15.6 ± 2	0.26
Age of onset, mean ± SD (years)	14.3 ± 3.4	14 ± 2.3	0.71
Age of diagnosis, mean ± SD (years)	14.9 ± 3.3	14.4 ± 2,2	0.54
Disease duration, mean ± SD (month)	16.3 ± 26.1	13.5 ± 15.4	0.54
EDSS, median (range)	1.5 (0–3.5)	2 (0–6)	0.003
Ped-MSSS, mean ± SD	5.2 ± 3.2	7.2 ± 2.9	0.005
Number of relapse pre *T*_0_, median (range)	1 (1–6)	2 (0–10)	0.02
ARR pre T_0_, median (range)	1 (0.1–4.4)	1 [0–5.2]	0.03
N° Previous DMT *T*_0_, median (range)	1 (0–2)	1 (0–2)	0.38
Previous DMT *T*_0_^a^			
Interferon, *N* (%)	14 (87)	25 (86)	0.06
Glatiramer acetate, *N* (%)	1 (6.5)		
Dimethyl fumarate, *N* (%)	1 (6.5)	3 (10)	
Mitoxantrone, *N* (%)		1 (4)	
Reason for switch			
Inefficacy, *N* (%)	6 (37)	21 (72)	0.02
Tollerability, *N* (%)	10 (63)	8 (28)	
Safety concerns, *N* (%)			
MRI lesion load^b^			
Low, *N* (%)	3 (12)	9 (17)	0.77
Medium, *N* (%)	12 (44)	21 (39)	
High, *N* (%)	12 (44)	24 (44)	
MRI contrast enhancement^c^			
Yes, *N* (%)	14 (58)	40 (78)	0.07
No, *N* (%)	10 (42)	11 (22)	
JCV status^d^			
Positive, *N* (%)	10 (53)	7 (15)	0.002
Negative, *N* (%)	9 (47%)	39 (85)	
SDMT T0, mean ± SD^e^	60.2 ± 9.2	49.4 ± 7.9	0.04

*DMT* Disease modifying treatment, *EDSS* Expanded disability status scale, Ped-*MSSS* Paediatric Multiple Sclerosis Severity Score, *SD* standard deviation, *SDMT* Symbol Digit Modality Test

(a) over total patients switching treatment; (b) data available for 81 patients; (c) data available for 75 patients; (d) data available for 65 patients (e) data available for 13 patients;

^*^Chi-squared, t test or Wilcoxon rank-sum as appropriate

### Clinical and MRI measures at T_1_

Overall, patients were followed-up for a mean follow-up time at T_1_ of 13.9 ± 3.8 months with no differences in follow-up time between F-POMS and N-POMS (*p* = 0.95). Between T0 and T1, the percentage of patients switching to another DMT was not different between F-POMS (3 out of 27 [11.1%]) and N-POMS (6 out of 57 [10.5%]) (*p* = 0.93). F-POMS mostly switched for inefficacy () compared with N-POMS, who mostly switched for safety concerns due to JCV antibody positivity (2 out of 3 F-POMS vs 5 out of 6, *p* = 0.13). Time to first DMT switch was not different between the two groups (*p* = 0.76).

Both F-POMS and N-POMS showed ARR reduction between T_0_ and T_1_ (F-POMS: 1 [0.1–4.4] vs 0 [0–0.1], *p* < 0.001; N-POMS: 1 [0–5.2] vs 0 [0–0.2], *p* < 0.001). Eight out of 27 F-POMS (30%) and 8 out of 57 (14%) N-POMS experienced at least one relapse between T_0_ and T_1_ (*p* = 0.09). Time to first relapse was not different between the two groups (*p* = 0.76). Two N-POMS patients switched to fingolimod after 6 and 11 months from T_0_ because of JCV sieroconversion. None of the F-POMS switched to natalizumab.

F-POMS did not show EDSS change between T_0_ and T_1_ (1.5 [0–3.5] vs 1.5 [0–2.5], *p* = 0.56). Conversely, N-POMS showed EDSS reduction between T_0_ and T_1_ (2 [0–6] vs 2 [0–6], *p* < 0.001). MRI data at T_1_ were available for 73 out of 81 patients. Five out of 21 (23.8%) F-POMS and 11 out of 52 (21.2%) N-POMS showed MRI activity between T_0_ and T_1_ (*p* = 0.80). Finally, only 1 out of 5 F-POMS performed a SDMT assessment at T_1_ showing thus changes were not assessed whereas 7 out of 8 N-POMS performed a SDMT assessment at T_1_ showing increase in the SDMT score (48.4 ± 8.1 vs 54.2 ± 5.7, *p* = 0.03).

### Propensity score matching analysis

After PS matching, we included 42 POMS patients in the analysis (21 F-POMS and 21 N-POMS). Groups were comparable for age (*p* = 0.96), gender (*p* = 0.52), ARR at T_0_ (*p* = 0.89), EDSS at T_0_ (*p* = 0.62), Ped-MSSS (*p* = 0.64) and number of previous DMT at T_0_ (p = 1.0). Clinical features of patients included in the analysis following PS-matching are reported in Table 2. The time to first relapse occurrence as well as time to DMT switch was not different between the two groups (*p* = 0.17 [see Fig. 1] and *p* = 0.85, respectively).

**Table 2:** Demographic and clinical features for patients included in the propensity-score matched analysis according to treatment allocation

	T0		*p* value*	T1			*p* value **	T2	*p* value***
	Fingolimod	Natalizumab		Fingolimod	*p* value**	Natalizumab		Natalizumab	
Subjects	27	57		27		57	–	42	–
Age at *T*_0_, mean ± SD	16.2 ± 2.7	15.6 ± 1.9	0.26						
Sex									
Female, *N* (%)	19 (70)	40 (70)	0.98	19 (70)	–	40 (70)	–	31 (16)	–
Male, *N* (%)	8 (30)	17 (30)		8 (30)	–	17 (30)	–	11 (84)	–
EDSS, median (range)	1.5 (0–3.5)	2 (0–6)	0.003	1.5 (0–2.5)	0.56	2 (0–6)	*p* < 0.001	2 (0–7)	0.15
ARR, median (range)	1 (0.1–4.4)	1 (0–5.2)	0.03	0 (0–0.1)	*p* < 0.001	0 (0–0.2)	*p* < 0.001	0 (0–0.005)	0.86
SDMT, mean ± SD	60.2 ± 9.2^a^	49.4 ± 7.9^b^	0.04	–	–	54.2 ± 5.7	0.03	55 ± 7.6	0.79

	T0-PS adjusted		*p* value *	T1-PS adjusted			*p* value **	T2-PS adjusted	*p* value ***
	Fingolimod	Natalizumab		Fingolimod	*p* value **	Natalizumab		Natalizumab	
Subjects	21	21		21	–	21	–	17	–
Age at *T*_0_, mean ± SD	15.9 ± 2.8	15.9 ± 1.8	0.96	–	–	–	–	–	–
Sex									
Female, *N* (%)	14 (67)	12 (57)	0.52	14 (67)	–	12 (57)	–	11 (65)	–
Male, *N* (%)	7 (33)	9 (43)		7 (33)	–	9 (43)	–	6 (35)	–
EDSS, median (range)	1.5 (0–3.5)	1.5 (0–3)	0.62	1.5 (0–2.5)	0.98	1 (0–2.5)	0.18	1.5 (0–7)	0.46
ARR, median (range)	1 (0.3–4.4)	1 (0.5–3.2)	0.89	0 (0–0.01)	< 0.001	0 (0–0.01)	< 0.001	0 (0–0.001)	0.52
Ped-MSSS, mean ± SD	5.7 ± 3.0	5.3 ± 2.9	0.64	–	–	–	–	–	–
SDMT, mean ± SD	60.5 ± 10.6^c^	48.3 ± 1.5^d^	0.11	–	–	51 ± 1.4	0.11	–	–

*EDSS* Expanded Disability Status Scale, *Ped-MSSS* Paediatric Multiple Sclerosis Severity Score, *SD* standard deviation, *SDMT* Symbol Digit Modality Test, *ARR* Annualizes Relapse Rate

^*^Chi-squared, t test or Wilcoxon rank-sum as appropriate

^**^Paired *t* test or Wilcoxon rank-sum as appropriate compared to T0

^***^Paired *t* test or Wilcoxon rank-sum as appropriate compared to T1

(a) data available for five patients; (b) data available for 8 patients; (c) data available for four patients; (b) data available for three patients

### Clinical and MRI measures at T_2_ for N-POMS

Forty-two out of 57 N-POMS patients were followed-up at T_2_ with a mean follow-up time of 66.1 ± 55.4 months. Between T_1_ and T_2_, 9 out of 42 (21%) N-POMS switched to another DMTs after mean time of 64.4 ± 39.4 months with 8 patients switching for safety concerns and 1 patients switching for inefficacy. Seven out of 42 (17%) N-POMS experienced at least one relapse between T_1_ and T_2_ after 71.5 ± 47.2 months. EDSS and ARR did not change between T_1_ and T_2_. MRI data at T_2_ were available for 37 out of 42 patients. Eight out of 37 (22%) N-POMS showed MRI activity between T_1_ and T_2_. Finally, we collected SDMT from 5 out of 7 N-POMS who already performed SDMT assessment at T_1_ and no changes were observed (*p* = 0.79). Over the follow-up none of the patient experienced any serious adverse event including PML, hospitalization due to relapse severity or infections.

## Discussion

In this study, we investigated the efficacy of fingolimod and natalizumab in POMS in a multicentre setting. We reported that overall natalizumab was prescribed to patients with much higher disease activity. Notwithstanding this bias in patient selection, over the follow-up both natalizumab and fingolimod were able to control the ARR as well as the EDSS, and SDMT. The beneficial effect of natalizumab on clinical outcomes was also confirmed longitudinally. However, if groups were balanced for disease severity at baseline, the two DMTs showed comparable efficacy profiles over time.

The first finding concerns the attitude of neurologists in prescribing natalizumab to POMS patients with greater disease activity, forecasting higher disability risks over the follow-up. Specifically, in our cohort, N-POMS showed higher relapse rate, physical and cognitive disability and mostly switched from previous DMTs due to inefficacy. Previous reports in AOMS have already shown that clinicians are usually more prone in prescribing natalizumab instead of fingolimod in highly active patients notwithstanding fingolimod shows a better ease of use (oral vs intravenous administration) and a lesser extent of life-threatening adverse event (i.e., progressive multifocal leucoencephalopathy) [16, 21]. In a recent registry-based study, the same attitude was also demonstrated for POMS and, hence, our study is in line with the report [27, 28]. However, the reason underpinning such attitude is still unclear. In clinical settings, natalizumab was proven to be more effective in controlling fatigue with a better patients’ perception vs fingolimod [28, 29]. Possibly, clinicians also consider these aspects and their impact on the on the overall disability, and, thus, tend to prescribe more often natalizumab in those patients with higher risk of disability accrual over the follow-up.

In addition, natalizumab was proven to be able to rapidly promote disability improvement after clinical relapses possibly through rapid resolution of acute inflammation allowing proper repair actions [30]. It was also demonstrated that delayed natalizumab start was associated with increases relapse frequency and motor disability [31–33]. These factors might provide rationale for selecting natalizumab over fingolimod treatment in POMS with more severe disease onset.

Another aspect that might drive clinicians to use natalizumab over fingolimod in patients with more severe disease activity is the treatment efficacy profile. In both adults and pediatric MS patients, previous studies evaluating treatment efficacy for fingolimod and natalizumab suggested an outperformance of the latter in halting disability accrual and disease flares over the follow-up [16–19].

However, these studies also highlight the difference for clinical and demographic baseline features for patients in the two groups, suggesting caution should be taken when interpreting or planning a randomized clinical trial. To overcome the lack of a randomized clinical trial, statistical tools could be applied to assess treatment efficacy in real-world setting, adjusting analyses for baseline features between groups. PS matching is the most frequently used among these tools allowing the definition of subgroups from larger groups with a balance for selected variables. This score has been widely applied in many therapeutic areas, including MS, to adjust for the uncontrolled assignment of treatment in observational studies [34]. As we already demonstrated in adults [16], when accounting for clinical features at baseline, natalizumab and fingolimod exert similar effect in modifying disease activity and, hence, could be used in a similar clinical landscape. Indeed, when evaluating the switch from platform therapies in patients with active disease, natalizumab is more effective than fingolimod in adult MS patients [35].

Finally, we also demonstrated the long-term efficacy of natalizumab in POMS. Specifically, natalizumab showed sustained efficacy in reducing disability accrual and relapse occurrence up to 5 years. Treatment with fingolimod was previously associated with favorable outcomes in adult- adult onset onset multiple sclerosis patients [36] as well as in POMS [37–40] as assessed through randomized clinical trials. On the other hand, we do not have proper randomized clinical trial assessing the efficacy of natalizumab vs fingolimod in POMS in the long run. This prevents natalizumab from being approved by regulatory agencies as treatment for POMS. To circumvent this shortage, several real-world studies highlighting the sustained efficacy of natalizumab are accumulating [12, 16–18, 27]. Therefore, to not necessarily prescribe natalizumab as an off-label treatment when considering natalizumab treatment as first line therapy in POMS, even below 12 years of age, our data would further support the drug-approval process from regulatory agencies. In addition, besides intravenous treatment, natalizumab could be prescribed as subcutaneous administration in adult patients after 1 year of intravenous infusion, reducing the burden for clinical facilities needed to administer infusion treatment.

We do acknowledge that this study is not without limitations. First, while this is a longitudinal study, given the retrospective nature of the study, previously collected data might not be complete and recorded in a systematic way. Furthermore, while the multicentre nature of the study allows the possibility of enrolling a larger sample size, this would also carry greater variability in data collection ad clinicians’ behavior in treatment prescription. Indeed, while the study is multicentre in nature, enrolling centres are tertiary centres where usually more severe cases are addressed. Hence, our findings might not reflect the attitude of clinicians toward less severe cases. Second, we relied on clinicians’ evaluation for radiologic report of the MRI activity without a centralized MRI assessment. Since no specific MRI protocols were defined, data might not be uniform throughout participating centres. Similarly, SDMT assessment was not a standard procedure for the study and, consequently, we only collected few data on the cognitive status of the patients. This led to the absence of valuable information on the effect of natalizumab and fingolimod on cognitive function in POMS. Finally, although PS is a powerful tool to correct for group differences in terms of clinic-demographic variables, differently from randomization, it might lack a proper group balance for unobserved variables.

In conclusion, in our study, we observed that clinicians usually prescribe natalizumab more frequently than fingolimod in POMS patients showing higher inflammatory activity. Natalizumab showed a greater effect in controlling relapses and reducing disability in POMS, although this effect seems to be mostly attributable to the high inflammatory activity of patients treated with natalizumab. Therefore, it might be valuable to select natalizumab over fingolimod in patients with more severe inflammatory activity. Indeed, when balancing group for disease-specific features, the two treatments showed the same efficacy profile. Finally, we reported that natalizumab showed a sustained beneficial effect in the long run, without safety concerns in POMS, thus, adding further finding in support of the approval process for the natalizumab treatment in young patients.

## References

[1] Jeong A, Oleske DM, Holman J (2019) Epidemiology of pediatric-onset multiple sclerosis: a systematic review of the literature. J Child Neurol 34:705–712. 10.1177/0883073819845827 31185780 10.1177/0883073819845827

[2] Renoux C, Vukusic S, Mikaeloff Y et al (2007) Natural history of multiple sclerosis with childhood onset. N Engl J Med 356:2603–2613. 10.1056/NEJMoa067597 17582070 10.1056/NEJMoa067597

[3] Yeh EA, Weinstock-Guttman B, Ramanathan M et al (2009) Magnetic resonance imaging characteristics of children and adults with paediatric-onset multiple sclerosis. Brain 132:3392–3400. 10.1093/brain/awp278 19892770 10.1093/brain/awp278

[4] Fadda G, Brown RA, Longoni G et al (2018) MRI and laboratory features and the performance of international criteria in the diagnosis of multiple sclerosis in children and adolescents: a prospective cohort study. Lancet Child Adolesc Health 2:191–204. 10.1016/S2352-4642(18)30026-9 30169254 10.1016/S2352-4642(18)30026-9

[5] Jakimovski D, Awan S, Eckert SP et al (2022) Multiple sclerosis in children: differential diagnosis, prognosis, and disease-modifying treatment. CNS Drugs 36:45–59. 10.1007/s40263-021-00887-w 34940954 10.1007/s40263-021-00887-w PMC8697541

[6] Ghezzi A, Deplano V, Faroni J et al (1997) Multiple sclerosis in childhood: clinical features of 149 cases. Mult Scler 3:43–46. 10.1177/135245859700300105 9160345 10.1177/135245859700300105

[7] Iaffaldano P, Portaccio E, Lucisano G et al (2024) Multiple sclerosis progression and relapse activity in children. JAMA Neurol 81:50–58. 10.1001/jamaneurol.2023.4455 38010712 10.1001/jamaneurol.2023.4455 PMC10682937

[8] Baroncini D, Simone M, Iaffaldano P et al (2021) Risk of persistent disability in patients with pediatric-onset multiple sclerosis. JAMA Neurol 78:726–735. 10.1001/jamaneurol.2021.1008 33938921 10.1001/jamaneurol.2021.1008 PMC8094039

[9] Ghezzi A, Amato MP, Edan G et al (2021) The introduction of new medications in pediatric multiple sclerosis: open issues and challenges. Mult Scler 27:479–482. 10.1177/1352458520930620 32539596 10.1177/1352458520930620

[10] Chitnis T, Arnold DL, Banwell B et al (2018) Trial of fingolimod versus interferon beta-1a in PEDIATRIC MULTIPLE SCLEROSIS. N Engl J Med 379:1017–1027. 10.1056/NEJMoa1800149 30207920 10.1056/NEJMoa1800149

[11] Saponaro AC, Tully T, Maillart E et al (2023) Treatments of paediatric multiple sclerosis: efficacy and tolerance in a longitudinal follow-up study. Eur J Paediatr Neurol 45:22–28. 10.1016/j.ejpn.2023.05.001 37245449 10.1016/j.ejpn.2023.05.001

[12] Baroncini D, Ghezzi A, Guaschino C et al (2022) Long-term follow-up (up to 11 years) of an Italian pediatric MS cohort treated with Natalizumab: a multicenter, observational study. Neurol Sci 43:6415–6423. 10.1007/s10072-022-06211-8 35781765 10.1007/s10072-022-06211-8

[13] Ghezzi A, Pozzilli C, Grimaldi LM et al (2010) Safety and efficacy of natalizumab in children with multiple sclerosis. Neurology 75:912–917. 10.1212/WNL.0b013e3181f11daf 20820002 10.1212/WNL.0b013e3181f11daf

[14] Determina (2020) n. 142638/2020. GU Serie Generale n.322

[15] Langer-Gould A, Atlas SW, Green AJ et al (2005) Progressive multifocal leukoencephalopathy in a patient treated with natalizumab. N Engl J Med 353:375–381. 10.1056/NEJMoa051847 15947078 10.1056/NEJMoa051847

[16] Lanzillo R, Carotenuto A, Moccia M et al (2017) A longitudinal real-life comparison study of natalizumab and fingolimod. Acta Neurol Scand 136:217–222. 10.1111/ane.12718 27976804 10.1111/ane.12718

[17] Koch-Henriksen N, Magyari M, Sellebjerg F et al (2017) A comparison of multiple sclerosis clinical disease activity between patients treated with natalizumab and fingolimod. Mult Scler 23:234–241. 10.1177/1352458516643393 27055806 10.1177/1352458516643393

[18] Boziki M, Bakirtzis C, Giantzi V et al (2021) Long-Term Efficacy Outcomes of Natalizumab vs. Fingolimod in Patients With Highly Active Relapsing-Remitting Multiple Sclerosis: Real-World Data From a Multiple Sclerosis Reference Center. Front Neurol 12:699844. 10.3389/fneur.2021.699844 34497577 10.3389/fneur.2021.699844 PMC8419322

[19] Cohen M, Mondot L, Bucciarelli F et al (2021) BEST-MS: a prospective head-to-head comparative study of natalizumab and fingolimod in active relapsing MS. Mult Scler 27:1556–1563. 10.1177/1352458520969145 33124504 10.1177/1352458520969145

[20] Prosperini L, Sacca F, Cordioli C et al (2017) Real-world effectiveness of natalizumab and fingolimod compared with self-injectable drugs in non-responders and in treatment-naive patients with multiple sclerosis. J Neurol 264:284–294. 10.1007/s00415-016-8343-5 27878443 10.1007/s00415-016-8343-5

[21] Andersen JB, Sharmin S, Lefort M et al (2021) The effectiveness of natalizumab vs fingolimod-A comparison of international registry studies. Mult Scler Relat Disord 53:103012. 10.1016/j.msard.2021.103012 34116480 10.1016/j.msard.2021.103012

[22] Krupp LB, Tardieu M, Amato MP et al (2013) International Pediatric Multiple Sclerosis Study Group criteria for pediatric multiple sclerosis and immune-mediated central nervous system demyelinating disorders: revisions to the 2007 definitions. Mult Scler 19:1261–1267. 10.1177/1352458513484547 23572237 10.1177/1352458513484547

[23] Kurtzke JF (1983) Rating neurologic impairment in multiple sclerosis: an expanded disability status scale (EDSS). Neurology 33:1444–1452. 10.1212/wnl.33.11.1444 6685237 10.1212/wnl.33.11.1444

[24] Santoro JD, Waltz M, Aaen G et al (2020) Pediatric Multiple Sclerosis Severity Score in a large US cohort. Neurology 95:e1844–e1853. 10.1212/WNL.0000000000010414 32690790 10.1212/WNL.0000000000010414 PMC7682820

[25] Scaravilli A, Tranfa M, Pontillo G et al (2024) Radiological Reporting Systems in Multiple Sclerosis. Appl Sci 14:5626

[26] Benedict RH, DeLuca J, Phillips G et al (2017) Validity of the Symbol Digit Modalities Test as a cognition performance outcome measure for multiple sclerosis. Mult Scler 23:721–733. 10.1177/1352458517690821 28206827 10.1177/1352458517690821 PMC5405816

[27] Spelman T, Simoneau G, Hyde R et al (2024) Comparative effectiveness of natalizumab, fingolimod, and injectable therapies in pediatric-onset multiple sclerosis: a registry-based study. Neurology 102:e208114. 10.1212/WNL.0000000000208114 38447093 10.1212/WNL.0000000000208114 PMC11033984

[28] Foley J, Berkovich R, Gudesblatt M et al (2023) Characterizing the “feel-good experience” in multiple sclerosis patients treated with natalizumab or other therapies. Neurodegener Dis Manag 13:23–34. 10.2217/nmt-2022-0003 36285716 10.2217/nmt-2022-0003

[29] Svenningsson A, Falk E, Celius EG et al (2013) Natalizumab treatment reduces fatigue in multiple sclerosis. Results from the TYNERGY trial; a study in the real life setting. PLoS ONE 8:e58643. 10.1371/journal.pone.0058643 23555589 10.1371/journal.pone.0058643 PMC3605436

[30] Belachew S, Phan-Ba R, Bartholome E et al (2011) Natalizumab induces a rapid improvement of disability status and ambulation after failure of previous therapy in relapsing-remitting multiple sclerosis. Eur J Neurol 18:240–245. 10.1111/j.1468-1331.2010.03112.x 20561044 10.1111/j.1468-1331.2010.03112.x

[31] Jafarpour S, Pinto S, Vu MH et al (2024) Delayed initiation of disease modifying therapy increases relapse frequency and motor disability in pediatric onset multiple sclerosis. Mult Scler Relat Disord 87:105669. 10.1016/j.msard.2024.105669 38749351 10.1016/j.msard.2024.105669

[32] Abdel-Mannan OA, Manchoon C, Rossor T et al (2021) Use of disease-modifying therapies in pediatric relapsing-remitting multiple sclerosis in the United Kingdom. Neurol Neuroimmunol Neuroinflamm. 10.1212/NXI.0000000000001008 34021056 10.1212/NXI.0000000000001008 PMC8143699

[33] Krysko KM, Graves JS, Rensel M et al (2020) Real-world effectiveness of initial disease-modifying therapies in pediatric multiple sclerosis. Ann Neurol 88:42–55. 10.1002/ana.25737 32267005 10.1002/ana.25737

[34] Trojano M, Pellegrini F, Paolicelli D et al (2009) Observational studies: propensity score analysis of non-randomized data. Int MS J 16:90–97 19878631

[35] Kalincik T, Horakova D, Spelman T et al (2015) Switch to natalizumab versus fingolimod in active relapsing-remitting multiple sclerosis. Ann Neurol 77:425–435. 10.1002/ana.24339 25546031 10.1002/ana.24339

[36] Cohen JA, Khatri B, Barkhof F et al (2016) Long-term (up to 4.5 years) treatment with fingolimod in multiple sclerosis: results from the extension of the randomised TRANSFORMS study. J Neurol Neurosurg Psychiatry 87:468–475. 10.1136/jnnp-2015-310597 26111826 10.1136/jnnp-2015-310597 PMC4853559

[37] Arnold DL, Banwell B, Bar-Or A et al (2020) Effect of fingolimod on MRI outcomes in patients with paediatric-onset multiple sclerosis: results from the phase 3 PARADIGMS study. J Neurol Neurosurg Psychiatry 91:483–492. 10.1136/jnnp-2019-322138 32132224 10.1136/jnnp-2019-322138 PMC7231437

[38] Borriello G, Pozzilli C (2021) Long-term fingolimod treatment in two pediatric patients with multiple sclerosis. Neurol Sci 42:29–36. 10.1007/s10072-021-05116-2 33751260 10.1007/s10072-021-05116-2

[39] Krupp L, Banwell B, Chitnis T et al (2022) Effect of fingolimod on health-related quality of life in paediatric patients with multiple sclerosis: results from the phase 3 PARADIGMS Study. BMJ Neurol Open 4:e000215. 10.1136/bmjno-2021-000215 35308898 10.1136/bmjno-2021-000215 PMC8883212

[40] Deiva K, Huppke P, Banwell B et al (2020) Consistent control of disease activity with fingolimod versus IFN beta-1a in paediatric-onset multiple sclerosis: further insights from PARADIGMS. J Neurol Neurosurg Psychiatry 91:58–66. 10.1136/jnnp-2019-321124 31467033 10.1136/jnnp-2019-321124 PMC6952840

